# Cognitive Impairment after Severe Traumatic Brain Injury, Clinical Course and Impact on Outcome: A Swedish-Icelandic Study

**DOI:** 10.1155/2015/680308

**Published:** 2015-12-09

**Authors:** Maud Stenberg, Alison K. Godbolt, Catharina Nygren De Boussard, Richard Levi, Britt-Marie Stålnacke

**Affiliations:** ^1^Department of Community Medicine and Rehabilitation, Rehabilitation Medicine, Umeå University, 901 85 Umeå, Sweden; ^2^Department of Clinical Sciences, Karolinska Institutet and University Department of Rehabilitation Medicine Stockholm, Danderyd Hospital, 182 88 Stockholm, Sweden

## Abstract

*Objective*. To assess the clinical course of cognitive and emotional impairments in patients with severe TBI (sTBI) from 3 weeks to 1 year after trauma and to study associations with outcomes at 1 year.* Methods*. Prospective, multicenter, observational study of sTBI in Sweden and Iceland. Patients aged 18–65 years with acute Glasgow Coma Scale 3–8 were assessed with the Barrow Neurological Institute Screen for Higher Cerebral Functions (BNIS) and the Hospital Anxiety and Depression Scale (HADS). Outcome measures were Glasgow Outcome Scale Extended (GOSE) and Rancho Los Amigos Cognitive Scale-Revised (RLAS-R).* Results*. Cognition was assessed with the BNIS assessed for 42 patients out of 100 at 3 weeks, 75 patients at 3 months, and 78 patients at 1 year. Cognition improved over time, especially from 3 weeks to 3 months. The BNIS subscales “orientation” and “visuospatial and visual problem solving” were associated with the GOSE and RLAS-R at 1 year.* Conclusion*. Cognition seemed to improve over time after sTBI and appeared to be rather stable from 3 months to 1 year. Since cognitive function was associated with outcomes, these results indicate that early screening of cognitive function could be of importance for rehabilitation planning in a clinical setting.

## 1. Introduction

The annual incidence of traumatic brain injury (TBI) in Sweden is estimated at 250–350 000/year [1, 2]. Severe traumatic brain injury (sTBI) defined by a Glasgow Coma Scale (GCS) of 3–8 [3] is much rarer, with incidence estimates of 4–8/100 000/year [1]. However, sTBI constitutes a major health problem due to the major functional impact of the injures and the individual suffering of patients and their families. Severe traumatic brain injuries comprise a heterogeneous group with varying complexity of deficits and impairments that may affect both physical and mental status. Over time, neurobehavioral and cognitive impairments have been shown to contribute more than physical impairments to the overall disability after TBI [4]. The patients' mental status is related to “disturbances in higher cerebral functions,” comprising not only cognition, but also the integration of cognition and emotion [5]. Dysfunction is reflected in a range of symptoms, such as frustration, inappropriate affective reactions, and lack of spontaneity [6].

Cognitive deficits after sTBI have been relatively well investigated. Most studies have focused on deficits in memory, processing speed, visual spatial abilities, and abstract reasoning [7]. However, the impact on affective functions as well as awareness during the early stages after brain injury has not been studied to the same extent. Borgaro et al. [8] investigated disturbances in affective communication in the acute stage in TBI-patients and patients with stroke. Both patient groups performance was significantly inferior to a control group as regards affect, expression, perception, and spontaneity. In a previous study by Prigatano and Wong [9], cognitive and affective impairments were shown to affect the achievement of rehabilitation goals in the early stages after TBI. Prigatano [10] states that the assessment of higher cerebral functions including both affective and cognitive deficits seems to be important for outcome after TBI. Neuropsychological examination is time-consuming and patients in the acute phase may be too ill to take part in a lengthy assessment. Therefore, in a clinical setting, there is a need for a brief cognitive screening instrument that can be easily used to establish a cognitive baseline that includes a range of higher cerebral functions in order to follow the patient's improvement and recovery over time.

The Barrow Neurological Institute Screen for Higher Cerebral Functions (BNIS) [11] is an instrument that may be used to screen both cognitive and affective disturbances, in particular in patients in the early posttraumatic stages of brain injury. The BNIS begins with three prescreen items that assess level of arousal, basic communication level, and level of cooperation. If the patient passes the prescreen, the BNIS can be completed. The instrument has been used in several studies of TBI [12], in which memory, awareness, and affect have been reported as among the most impaired functions [6, 8].

Emotional disturbances, including symptoms of depression and anxiety, are a major cause of disability after TBI and comprise risk factors for poor recovery [5]. It is now recommended that patients undergoing rehabilitation following TBI should be assessed for mood disorders [13]. However, in studies on the prevalence of depression and anxiety in TBI patients the results vary widely. One possible reason may be related to different methods of assessment. There are several rating scales that can be used while other researchers may prefer structured interviews. One of the most common instruments used both in the literature and in clinical health care is the Hospital Anxiety and Depression Scale (HADS) [14]. It has been studied in a large number of different patient groups and has been suggested as a screening instrument of choice for anxiety and depression [15]. Since cognitive, affective, and emotional impairments all may play an important role in recovery after sTBI, we decided to study these factors during the first year after sTBI in adults admitted to neurosurgical departments as part of a large prospective multicentre cohort study (“PROBRAIN”) [16–18]. Previous mental illness and cognitive problems may affect the patients' health and their posttrauma condition; we therefore considered that these factors were also of importance to investigate. Patients with such preexisting problems may also be more prone to TBI [19], so excluding this group would limit generalizability.

The aims of this study were to assess the clinical course of cognitive and emotional impairments as assessed by BNIS and by HADS from three weeks to one year after trauma and to study associations with outcomes at one year.

## 2. Methods

### 2.1. Patients

Inclusion criteria were as follows: (1) patients with severe TBI, who survived at least 3 weeks with a lowest nonsedated GCS [3] 3–8 or equivalent scores on the Swedish Reaction Level Scale (RLS) [20] in the first 24 hours after injury and who were able to complete a brief screening test with BNIS prescreening, were then assessed with the full BNIS test; (2) age at injury: 18–65 years; (3) injury requiring neurosurgical intensive care or collaborative care with a neurosurgeon and a physician in another intensive care unit; (4) patients were required to speak and understand the Swedish or Icelandic language. Exclusion criteria were death or expected death within 3 weeks of injury.

The 8-point RLS is widely used in Sweden in some emergency departments and neurosurgical units instead of the GCS. The RLS criteria were therefore necessary to allow recruitment of patients from the centers using this scale. Scores on the GCS of 3–8 and on the RLS of 8–4 reflect similar severity of injury [21]. RLS scoring is in the opposite direction to GCS scoring, with the highest RLS score of 8 reflecting the most severe injuries (GCS 3).

### 2.2. Procedure

Patients from neurosurgical intensive care units at five neurotrauma centres in Sweden and one in Iceland were included. Patients were recruited prospectively by rehabilitation physicians from January 2010 until June 2011, with extended recruitment until December 2011 at 2 centres. The participating centres provide neurosurgical care to >80% of the respective populations of Sweden and Iceland. Patients were evaluated at 3 weeks, 3 months, and 1 year after injury. The patient gave written informed consent in cases where he or she had the capacity to do so. In the majority of cases, the patient lacked that capacity and the patient's nearest relative gave consent to inclusion. Assessments took place in the patient's current care setting or in a local outpatient department. Inclusion and follow-up were therefore independent of the patient's clinical course and care setting. Assessments were performed by rehabilitation physicians with assistance from rehabilitation nurses, psychologists, physiotherapists, and occupational therapists. The data regarding education and earlier cognitive problems were obtained by interviews of patients and/or significant others.

Patients were interviewed and administered the Barrow Neurological Institute Screen for Higher Cerebral Functions (BNIS), either by a clinical neuropsychologist or a physician who was a specialist in rehabilitation medicine. Prescreening was performed initially to evaluate whether it was meaningful to attempt further testing. The BNIS takes about 10–25 minutes to complete.

### 2.3. Instruments

#### 2.3.1. The Barrow Neurological Institute Screen for Higher Cerebral Functions (BNIS)

The BNIS (11) is a cognitive screening test for speech and language functions, orientation, attention/concentration, visuospatial and visual problem solving, memory, affect, and awareness. The BNIS test comprises a prescreen test (level of arousal 3 p, basic communication 3 p, and cooperation 3 p). The three items in BNIS prescreening must be assessed and the patients must achieve at least two points on each of the items for it to be meaningful to continue. Lower scores indicates that the patient will not be able to perform the BNIS. BNIS yields a total score and seven subscale scores. The total score (maximum 50 points) consists of the results from the prescreen plus the 7 subscale scores (speech and language 15 p, orientation 3 p, attention/concentration 3 p, visuospatial and visual problem solving 8 p, memory and learning 7 p, affect (generating happy versus angry affect, perception of facial affect, affect control, and ability to generate spontaneity) 4 p, and awareness of own performance 1 p). A total subscale score can be obtained, as well as a total BNIS raw score that is converted to an age-corrected standard *T*-score. Higher scores reflect a higher level of functioning. If the total BNIS score is below 47 points, further cognitive investigation is recommended [22]. The BNIS has been validated for a Swedish population [23, 24]. The BNIS was assessed at 3 weeks, 3 months, and 1 year after injury.

#### 2.3.2. The Hospital Anxiety and Depression Scale (HADS)

The Hospital Anxiety and Depression Scale (HADS) [14] was used to screen for presence and degree of anxiety and depression. It consists of 14 items (7 items in each subscale) which are assessed on a 4-point Likert scale (range 0–3), where the total score is the sum of each subscale (range 0–21). Cut-offs for both subscales of 8 or higher were used to determine “caseness” [25]. The HADS is an established screening tool for anxiety and depression and it has been used previously in patients with TBI [13]. The HADS has acceptable reliability, sensitivity, and specificity in assessing symptom severity in anxiety and depression in various populations [26]. The HADS was assessed at 3 weeks, 3 months, and at 1 year after injury.

#### 2.3.3. Outcomes

Outcome variables were Glasgow Outcome Scale Extended (GOSE) [27] and Rancho Los Amigos Cognitive Scale-Revised (RLAS-R). The RLAS-R was used at 3 weeks, 3 months, and 1 year and the GOSE at 1 year.

#### 2.3.4. The Glasgow Outcome Scale Extended (GOSE)

The Glasgow Outcome Scale Extended (GOSE) [27] extends the 5 categories of the previously developed Glasgow Outcome Scale (GOS) [28] to 8, thereby increasing its sensitivity. The 8 categories span from “Dead” (score 1) to “Upper Good Recovery” (score 8). For those alive at one year, GOSE was dichotomized into “Unfavourable outcome” (GOSE 1–4) and “Favourable outcome” (GOSE 5–8). The GOSE has good interrater reliability [27] and validity [29] and is an established measure of global outcome after traumatic brain injury.

#### 2.3.5. Rancho Los Amigos Scale of Cognitive Functioning-Revised (RLAS-R)

To enable our findings to be considered in relation to phase of recovery after sTBI, the Rancho Los Amigos Scale of Cognitive Functioning (RLAS-revised) [30] was assessed. RLAS-R is a clinical scale with scores from 1 to 10, representing ten states of cognitive and behavioral functioning through which patients with TBI typically progress (see Table 1). Higher scores indicate improved functioning. The lowest level is “No Response, Total Assistance,” and the highest level is “Purposeful, Appropriate: Modified Independent.”

The RLAS originally had 8 levels, while the revision added levels 9 and 10 to better reflect the highest levels of recovery. The original levels and the revised levels of the RLAS-R levels were dichotomized into two categories: “inferior functioning” (RLAS-R 1–8) and “superior functioning” (RLAS-R 9-10).

### 2.4. Statistical Analysis

Data were analyzed with SPSS, version 21.0 for Windows. Data were reported as frequencies or median and IQR and means. Nonparametric tests were used as the samples were small and/or not normally distributed. Thus, the Mann-Whitney test was used for the comparison of continuous variables and Wilcoxon's signed rank test for the study of paired observation variables. The Spearman correlation coefficient was used for the analysis of bivariate correlation. The Chi-square test was used for the comparison of proportions. The level of statistical significance was set as *p* = 0.01. Univariate binary logistic regression analyses were performed where statistically appropriate, to explore associations between the BNIS raw scores with outcome. Variables found to be significant (*p* < 0.05) with univariate analyses were incorporated into a multivariate model using a forward method, with a cut-off for rejection of variables from the model of *p* = 0.10.

## 3. Results

### 3.1. Patient Characteristics

**Figure 1:**
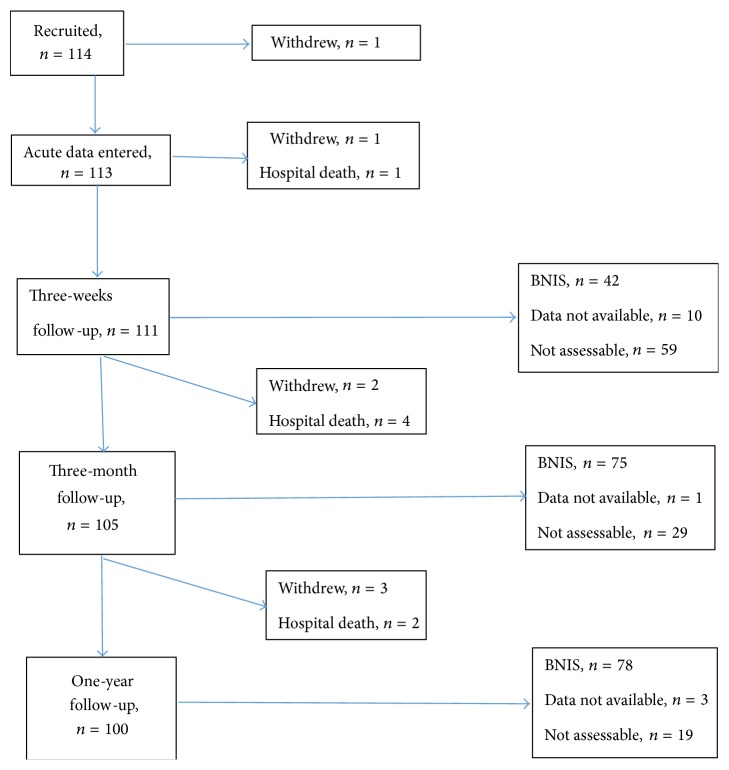
Flow chart of patients through the study.

Figure 1 is a flow chart depicting the study process. One hundred and fourteen patients were recruited and 78 completed the BNIS at 1 year after trauma. Seven patients died during follow-up (1 before the 3-week assessment, another 4 before the 3-month assessment, and 2 after that). A further 7 patients withdrew (2 before the 3-week assessment, another 2 before the 3-month assessment, and 3 after that). Basic patient descriptors are shown in Table 2. Patients who died were older (median age 61 years, range 19–64) and had lower acute GCS (median 3, range 3–7) while patients who withdrew were younger (median age 32.5 years, range 20–56) and had higher median GCS 6 (3–7). The median age of patients who participated in the study was 42 years (range 17–65), and GCS during the first 24 hours was median 5 (3–8). One patient was included shortly before the patient's 18th birthday due to a minor protocol violation. Eighty-six were men and 28 were women. Less than 12 years of education was reported by 35 men (33%) and 10 women (9%).

### 3.2. The Barrow Neurological Institute Screen for Higher Cerebral Functions (BNIS)

See Table 3. Three weeks after injury, the BNIS could be assessed in 42 patients, 59 patients could not be assessed due to ongoing disorders of consciousness (DOC) or sedation, and data were missing for 10 patients. Out of the 59 nonassessed patients, 7 patients were assessed with the prescreen at 3 weeks but they did not reach the level to perform the BNIS. At 3 months, 75 patients were assessed and 29 patients could not be assessed. There were missing data for 1 patient. At 1-year follow-up, 78 patients were assessed, 19 patients could not be assessed, and data were missing for 3 patients. Out of the 19 nonassessed patients, 8 patients were assessed with the BNIS prescreen at 1 year and scored too low for administration of the BNIS to be possible. Both the BNIS total raw scores and *T*-scores improved significantly from 3 weeks to 3 months after injury (raw score: *p* < 0.001, *T*-score: *p* < 0.001) and from 3 months to 1 year on the raw score only (*p* = 0.004) and *T*-score (*p* = 0.086). The total subscales scores were significantly improved from 3 weeks to 3 months (*p* < 0.001) and from 3 months to 1 year close to significant (*p* = 0.015). Significant improvement on the separate BNIS subscales was shown from 3 weeks to 3 months for speech/language (*p* = 0.002) and memory (*p* = 0.001). From 3 months to 1 year, no further significant improvements were found. See Table 4.

When patients with more than 12 years of education were compared with patients with less than 12 years of education at 3 weeks, patients with the higher educational level had higher scores but the differences were nonsignificant. At 3 months and 1 year, patients with more than 12 years of education had statistically significant higher scores on the subscales speech/language (3 months = 0.001, 1 year: *p* < 0.001), orientation (3 months *p* = 0.002, 1 year: 0.001), attention/concentration (3 months: *p* = 0.002, 1 year: *p* = 0.004), visuospatial and visual problem solving (3 months: *p* = 0.002, nonsignificant at 1 year), memory (3 months = 0.002, 1 year: 0.005), affect (3 months = 0.001, 1 year = 0.001), and awareness (3 months *p* = 0.005, 1 year *p* = 0.005). The distribution of patients above and below the cut-off for cognitive dysfunction at 47 points and the corresponding levels for *T*-scores (22) are shown in Table 5.

### 3.3. The Hospital Anxiety and Depression Scale (HADS)

No statistically significant differences were found for HADS anxiety from 3 weeks to 3 months (*p* = 0.865) and from 3 months to 1 year (*p* = 0.702), nor for HADS depression from 3 weeks to 3 months (*p* = 0.915) and from 3 months to one year (*p* = 0.394). Scores above cut-off for HADS anxiety occurred in 16 of 75 assessable patients at 3 months and in 16 of 74 patients at 1 year after injury. HADS depression scores above cut-off occurred in 11 of 75 assessable patients at 3 months and in 15 of 74 patients at 1 year. Significant correlations were found between HADS depression and BNIS total at 3 months (*r* = −0.302, *p* = 0.009) and at one year (*r* = −0.361, *p* = 0.002). No statistically significant correlation was found between HADS anxiety and BNIS at 3 months and 1 year.

### 3.4. Outcome as Assessed with the Glasgow Outcome Scale Extended (GOSE)

Patients who completed the BNIS at 3 weeks, 3 months, and 1 year, and with “favourable” and “unfavourable” outcomes, respectively, on the GOSE are shown in Table 6. Univariate logistic regression analyses demonstrated that the following variables at 3 months were associated with “favourable outcome” on the GOSE at 1 year: BNIS total scores (OR = 1.200, CI: 1.072–1.343, *p* = 0.002) and BNIS subscales orientation (OR = 4.177, CI: 1.850–9.430, *p* = 0.001), visuospatial and visual problem solving (OR = 2.156, CI: 1.371–3.391, *p* = 0.001), memory (OR = 1.492, CI: 1.084–2.052, *p* = 0.014), affect (OR: 2.910, CI: 1.483–5.713, *p* = 0.002), and awareness (OR = 5.714, CI: 1.153–28.322, *p* = 0.033). The subscales were incorporated into a multivariate model. The analysis showed that statistically significant associations were obtained for orientation (OR: 2.762, CI: 1.140–6.695, *p* = 0.024) and visuospatial and visual problem solving (OR: 1.930, CI: 1.181–3.155, *p* = 0.009). No significant association was found between HADS anxiety and HADS depression at 3 months and GOSE (anxiety OR = 0.954, CI: 0.409–2.226, *p* = 0.914) and depression (OR = 0.717, CI: 0.233–2.211, *p* = 0.563).

### 3.5. Outcome as Assessed with the Rancho Los Amigos Scale of Cognitive Functioning-Revised (RLAS-R)

Patients who completed the BNIS at 3 weeks, 3 months, and 1 year and with “superior” and “inferior functioning” on the RLAS-R are shown in Table 6. Univariate logistic regression analyses demonstrated that the following variables 3 months after injury were associated with “superior functioning” at 1 year: BNIS total scores (OR = 1.218, CI: 1.090–1.362, *p* = 0.001), BNIS subscales orientation (OR = 4.480, CI: 1.974–10.165, *p* < 0.001), visuospatial and visual problem solving (OR = 2.476, CI: 1.527–4.017, *p* < 0.001), memory (OR = 1.502, CI: 1.124–2.006, *p* = 0.006), affect (OR = 2.812, CI: 1.482–5.335, *p* = 0.002), and awareness (OR = 5.167, CI: 1.309–20.309, *p* = 0.019). In a multivariate model, statistically significant associations were obtained for orientation (OR = 3.325, CI: 1.298–8.519, *p* = 0.012) and visuospatial and visual problem solving (OR = 2.336, CI: 1.371–3.980, *p* = 0.002). No significant association was found between HADS anxiety and depression at 3 months and GOSE.

## 4. Discussion

This study shows that it is feasible to use the BNIS instrument for the screening of cognitive functions in a significant minority of patients (42%) as early as 3 weeks after severe traumatic brain injury. Such a screening has the potential to allow individualization of rehabilitation interventions at the stage of recovery where neuroplasticity is maximal, with potential outcome benefits.

In accordance with previous studies, the majority were males [1, 31]. The severity of TBI on the acute GCS (median GCS) was consistent with other prospective studies of sTBI [6]. However, it should be noted that the patients who died were older and had lower GCS while the patients who withdrew were younger and were less severely injured according to the GCS. Falls are a common cause of TBI in children and elderly persons [1, 31] and were also the most frequent cause in our population of working aged adults. Transport accidents were the second most common cause. Similar findings of falls causing most of TBI have been reported in some previous Scandinavian studies [1, 31] while motor vehicle injuries dominate in American [32] and Australian [33] studies.

The BNIS scores of the patients who completed the test at 3 weeks improved substantially at 3 months and further improvement was shown at 1 year. This is in keeping with clinical rehabilitation experience and highlights the importance of avoiding hasty decisions regarding discharge destination (nursing home or own home) and continued rehabilitation interventions based on overinterpretation of early cognitive performance. However, the number of patients who were able to complete the BNIS was relatively stable from 3 months to 1 year, such that few very severely injured patients who could not complete the BNIS at 3 months improved to a level where this could be completed at one year. When the BNIS total scores at 3 weeks were compared with the results reported by Borgaro and Prigatano [6] of a small population of sTBI patients early after the injury (around 20 days), the patients in our study performed better and the scores were even higher than a group of patients with moderate TBI, but lower than a control group. This finding can in part be explained by differences regarding study populations and a large variation in the ranges of postinjury time in the Borgaro and Prigatano study [6]. Although the BNIS-scores improved over time in our study, the scores at 1 year were in a range that was similar to that reported by Swedish TBI-patients from a neurorehabilitation clinic [24] indicating that the long-term results are probably relatively consistent. However, it is worth remembering that the BNIS is a screening instrument that can be used to detect patients in need of comprehensive cognitive neuropsychological assessment. According to the Swedish BNIS manual [22], the majority of our assessed patients at all the time points gained scores that were below the cut-off (less than 47 points) which means that they recommended further testing, but this proportion decreased over time from 84% to 74%.

About 35–40% of the patients in our study reported an education level of less than 12 years. In a recent Swedish study by Hofgren et al. [24], the BNIS in patient groups from a neurorehabilitation clinic was validated. An education level of less than 9 years was considered as being low since 9 years is compulsory in the Swedish educational system. In our study, the level of low education was chosen as lower than 12 years because the majority of the Swedish population continue to study at upper secondary school. Regardless of where the education level limit is set, it seems that the results in our study confirm prior results of a relationship between education level and cognition [6, 24]. In a clinical context, it is important to consider this in order to optimize the setting of realistic rehabilitation goals for each individual patient.

When comparing the scores of the subscales at the different time points, significant improvements in our study were only shown from 3 weeks to 3 months. The results at 3 months and at 1 year were in line with the previous Swedish results by Hofgren et al. [24]. Moreover, the majority of patients who completed the BNIS at all the three time points experienced “favourable outcome” on the GOSE and “superior functioning” on the RLAS. Higher scores on the orientation and visuospatial and visual problem solving subscales at 3 months were also associated with good outcomes.

Disorientation, a key component of posttraumatic amnesia, has often been studied in patients in the acute phase after TBI and has been reported as a predictor of cognitive impairments after injury [34]. Borgaro et al. [12] examined the utility of the BNIS to assess orientation in patients with TBI and concluded that the instrument was shown to be a sensitive measure of disorientation in these patients. Both the orientation and the visuospatial and visual problem solving subscales include basic domains of importance for independence inside and outside the patients' homes. It was therefore not surprising that these subscales were associated with outcome in the present study. Although the orientation and visuospatial problems could possibly have contributed to unfavourable outcome on the GOSE, there are also other causes of disability after sTBI, such as mental fatigue and executive dysfunctions. In a previous Swedish TBI-study, orientation and awareness on the BNIS were found to be two of the most common cognitive dysfunctions perceived as problems [24]. In our study, awareness on the BNIS subscale was associated to the GOSE. This result is in line with earlier studies which have reported a relationship between self-awareness and long-term outcome in TBI-patients [35]. In a study by Kelley et al. [36], impaired awareness was shown more than 5 years after TBI and awareness of cognitive function was found to predict return to work. Although awareness may improve over time, it seems to be a complex construct including varying aspects.

Studies have reported depression and anxiety as a major cause of disability after TBI [5, 37]. In the present study, there were negative relationships between the BNIS total score and the HADS anxiety and depression scores at the one-year follow-up, indicating that patients with a cognitive dysfunction may also suffer from anxiety and depression symptoms over time. These findings confirm earlier results which have shown an association between self-reported depression and anxiety and poor performance on cognitive tests [38]. In a rehabilitation context, these results imply the importance of screening cognitive difficulties and depression and anxiety to identify those who should be further assessed.

Most prior studies using the BNIS have used heterogeneous study populations with a mix of diagnoses, different TBI grades, and small TBI populations [8, 12, 24] which were studied at different time points after the trauma. Our study has several strengths, such as a prospective design and a large and well-characterized multicentre study population of patients with sTBI. In addition, the follow-up and the BNIS testing were performed by experienced staff, a clinical neuropsychologist, or physician working in rehabilitation medicine. The follow-up rate of 69% completing the BNIS is satisfactory. Only 19 patients could not complete the BNIS at the 1-year follow-up and data were missing for three patients. Since the incidence of sTBI is low in comparison with moderate and mild TBI [1], the population size of 114 patients in our prospective multicentre study could be considered relatively high. In addition, the low number of missing data also strengthens the study.

However, our study is based on a clinical population and has some limitations. Although we had weekly contact with intensive care units, we cannot exclude that some patients were admitted and discharged between contacts and would therefore have been missed from the recruitment process. Some data was also missing which may be due to this multicentre study design that included many assessment instruments with follow-up of patients from a wide geographical area in Sweden.

## 5. Conclusion

The results indicate that cognition improves over time after sTBI and appears to be relatively stable from 3 months to 1 year. Since cognitive function was associated with outcomes, it seems that early screening of cognitive function could be of importance for rehabilitation planning in a clinical setting.

## Figures and Tables

**Table 1:** Rancho Los Amigos Cognitive Scale-Revised, levels of cognitive functioning (RLAS-R).

Level	
I	No Response: Total Assistance
II	Generalized Response: Total Assistance
III	Localized Response: Total Assistance
IV	Confused/Agitated: Maximal Assistance
V	Confused, Inappropriate Non-Agitated: Maximal Assistance
VI	Confused, Appropriate: Moderate Assistance
VII	Automatic, Appropriate: Minimal Assistance for Daily Living Skills
VIII	Purposeful, Appropriate: Stand-By Assistance
IX	Purposeful, Appropriate: Stand-By Assistance on Request
X	Purposeful, Appropriate: Modified Independent

**Table 2:** Patient descriptors (*n* = 114).

Age at injury, years, median (range)		42 (17–65)

Gender (female/male)		(28/86)

Worst unsedated GCS^1^ 3–8 first 24 hours median, (range)		5 (3–8)

Cause of injury, *n* (%)	Transport accident	46 (41)
	Fall	50 (44)
	Other	13 (11)
	Missing	5 (4)

Length of stay in intensive care, days, median (range)		17 (1–78)

Economic support at time of injury, *n* (%)	Employed/self-employed fulltime	57 (50)
	Study grant	7 (6)
	Unemployment benefit or social support	11 (10)
	Sick pay	16 (14)
	Other^2^	8 (7)
	Part-time employment/self-employment	6 (5)
	Unknown	3 (3)
	Missing data	6 (5)

		*n*/total *n* (%)

Education <12 years, *n* = 107,		
missing = 7	Female	10/107 (9)
	Male	35/107 (33)
Previous brain injury requiring hospitalization, *n* = 105,		
missing = 9	Female	5/105 (5)
	Male	13/105 (12)
Previous brain injury requiring CT scan of the brain, *n* = 105,		
missing = 9	Female	6/105 (6)
	Male	12/105 (11)
Previous mental illness, *n* = 109,		
missing = 5	Female	6/109 (6)
	Male	12/109 (11)
Previous learning problem, *n* = 108,		
missing = 6	Female	3/108 (3)
	Male	9/108 (8)
Previous memory problems, *n* = 108,		
missing = 6	Female	3/108 (3)
	Male	12/108 (11)
Previous difficulty concentrating, *n* = 108,		
missing = 6	Female	3/108 (3)
	Male	17/108 (16)

^1^Conversion from RLS scores to GCS scores for those patients not assessed with GCS (*n* = 42).

^2^Other economic support.

**Table 3:** BNIS total (raw) score at 3 weeks, 3 months, and 1 year and description of educational level and previous medical disorders (including any learning, memory, or concentration difficulties).

3 weeks	Number (female/male)		42 (10/32)
	Total (raw**)** score median (range)		38.5 (18–50)
	Total (raw) score mean (SD)		37.2 (8.3)
	Education <12 years total (raw) score mean (SD)		34.8 (7.9)

			*n*/total *n* (%)

3 weeks	Education <12 years	Missing = 1	5 female + 14 male/41 (46)
	Previous brain injury requiring hospitalization	Missing = 3	6/39 (15)
	Previous brain injury requiring CT scan of the brain	Missing = 0	6/42 (14)
	Previous mental illness	Missing = 2	6/40 (15)
	Previous learning problem	Missing = 2	6/40 (15)
	Previous difficulty to remember	Missing = 2	8/40 (20)
	Previous difficulty to concentrate	Missing = 2	7/40 (17)

3 months	Number (female/male)		75 (19/56)
	Total (raw) score median (range)		41.0 (23–50)
	Total (raw) score mean (SD)		40.4 (7.0)
	Education <12 years, total (raw) score mean (SD)		38.2 (6.9)

			*n*/total *n* (%)

3 months	Education <12 years	Missing = 4	7 female + 22 male/71 (41)
	Previous brain injury requiring hospitalization	Missing = 8	12/67 (18)
	Previous brain injury requiring CT scan of the brain	Missing = 7	12/68 (18)
	Previous mental illness	Missing = 4	11/71 (15)
	Previous learning problem	Missing = 4	7/71 (10)
	Previous difficulty remembering	Missing = 4	10/71 (14)
	Previous difficulty concentrating	Missing = 4	11/71 (15)

1 year	Number (female/male)		78 (21/57)
	Total (raw) score median (range)		42.0 (16–50)
	Total (raw) score mean (SD)		40.3 (7.8)
	Education <12 years, total (raw) score mean (SD)		38.1 (7.6)

			*n*/total *n* (%)

1 year	Education <12 years	Missing = 4	7 female + 25 male/74 (43)
	Previous brain injury requiring hospitalization	Missing = 8	13/70 (19)
	Previous brain injury requiring CT scan of the brain	Missing = 8	12/70 (17)
	Previous mental illness	Missing = 4	13/74 (18)
	Previous learning problem	Missing = 4	7/74 (10)
	Previous difficulty to remember	Missing = 4	9/74 (12)
	Previous difficulty to concentrate	Missing = 4	12/74 (16)

**(a):** 

BNIS score 3 weeks (*n* = 42)			
Data not available (*n* = 10)	Mean (SD)	Median	Range
Not assessable (*n* = 59)			
Speech/language	12.4 (2.6)	14.0	5–15
Orientation	2.3 (0.8)	2.5	1–3
Attention/concentration	1.6 (1.1)	2.0	0–3
Visuospatial and visual problem solving	5.1 (1.9)	5.0	1–8
Memory	3.4 (2.3)	3.0	0–7
Affect	3.0 (1.2)	3.0	0–4
Awareness	0.4 (0.5)	0.0	0-1
Total BNIS (raw) score	37.4 (8.3)	38.5	18–50
Total *T*-score conversion	16.6 (19.6)	8.0	0.9–63

**(b):** 

BNIS score 3 months (*n* = 75)				3 weeks versus
Data not available (*n* = 1)	Mean (SD)	Median	Range	3 months
Not assessable (*n* = 29)				*p* value
Speech/language	13.4 (2.0)	14.0	7–15	0.002
Orientation	2.6 (0.7)	3.0	1–3	0.032
Attention/concentration	2.1 (0.7)	2.0	1–3	0.054
Visuospatial and visual problem solving	5.5 (1.9)	6.0	1–8	0.013
Memory	4.2 (2.3)	4.0	0–7	0.001
Affect	3.3 (0.9)	4.0	0–4	0.051
Awareness	0.5 (0.5)	0.0	0-1	0.860
Total BNIS (raw) score	40.4 (7.0)	41.0	23–50	<0.001
Total *T*-score conversion	25.1 (22.5)	23.0	0.9–63	<0.001

**(c):** 

BNIS 1 year (*n* = 78)				3 months versus
Data not available (*n* = 3)	Mean (SD)	Median	Range	1 year
Not assessable (*n* = 19)				*p* value
Speech/language	13.1 (2.7)	14.0	2–15	0.322
Orientation	2.7 (0.6)	3.0	1–3	0.072
Attention/concentration	2.0 (1.0)	2.0	1–3	0.682
Visuospatial and visual problem solving	5.5 (0.9)	6.0	1–8	0.136
Memory	4.3 (2.3)	4.0	0–7	0.082
Affect	3.3 (1.0)	4.0	0–4	0.870
Awareness	0.5 (0.5)	0.5	0-1	0.712
Total BNIS (raw) score	40.3 (7.8)	42.0	16–50	0.004
Total *T*-score conversion	25.1 (22.4)	23.0	0.9–63	0.086

**Table 5:** BNIS total score and *T*-score conversion, cut-off level of cognitive function (*n* = 114).

Total score	Points	3 weeks	3 months	1 year
		*n* (%)	*n* (%)	*n* (%)
Low probability of cognitive dysfunction	≥47	6 (16)	18 (24)	20 (26)
Recommendation of further investigation of cognitive function	<47	36 (84)	57 (76)	58 (74)
Total		42 (100)	75 (100)	78 (100)

Total *T*-score conversion		*n* (%)	*n* (%)	*n* (%)

Very low probability of cognitive dysfunction	>50	4 (7)	14 (19)	14 (18)
Low probability of cognitive dysfunction	40–50	3 (7)	9 (12)	11 (14)
Increased likelihood of cognitive dysfunction	30–39	3 (7)	10 (14)	10 (13)
High probability of cognitive dysfunction	<30	32 (76)	41 (55)	42 (54)
Missing			1 (1)	1 (1)
Total		42 (100)	75 (100)	78 (100)
Missing, *n* (% of all included)				
Data not available		10 (9)	1 (1)	3 (3)
Not assessable		59 (52)	29 (25)	19 (16)
Withdrew		2 (2)	4 (4)	7 (6)
Hospital death		1 (1)	5 (4)	7 (6)

**Table 6:** Patients who completed the BNIS total score and outcomes GOS-E and RLAS-R at 3 weeks, 3 months and 1 year.

		*N* (%)	Mean BNIS *T* score
BNIS 3 weeks (*n* = 42)	GOSE at 1 year		
	Unfavourable GOS-E 1–4	3 (7)	0.9
	Favourable GOS-E 5–8	35 (83)	19.1
	Missing	4 (10)	
	RLAS-R at 1 year		
	Inferior functioning	4 (10)
	RLAS-R 1–8	
	Superior functioning	34 (81)
	RLAS-R 9-10	
	Missing	4 (10)	

BNIS 3 month (*n* = 75)	GOSE at 1 year		
	Unfavourable GOS-E 1–4	12 (16)	8.8
	Favourable GOS-E 5–8	60 (80)	28.1
	Missing	3 (4)	
	RLAS-R at 1 year		
	Inferior functioning	15 (20)
	RLAS-R 1–8	
	Superior functioning	55 (73)
	RLAS-R 9-10	
	Missing	5 (7)	

BNIS 1 year (*n* = 78)	GOS-E 1 year		
	Unfavourable GOS-E 1–4	18 (23)	9.7
	Favourable GOS-E 5–8	59 (76)	29.9
	Missing	1 (1)	
	RLAS-R at 1 year		
	Inferior functioning	21 (27)
	RLAS-R 1–8	
	Superior functioning	54 (69)
	RLAS-R 9-10	
	Missing	3 (4)	
